# *AmpC β*-Lactamase Variable Expression in Common Multidrug-Resistant Nosocomial Bacterial Pathogens from a Tertiary Hospital in Cairo, Egypt

**DOI:** 10.1155/2021/6633888

**Published:** 2021-03-28

**Authors:** Aliaa Ali El Shamy, Zainab Zakaria, Mahmoud M. Tolba, Nermeen Salah Eldin, Al-Taher Rabea, Mahmoud M. Tawfick, Hebatallah A. Nasser

**Affiliations:** ^1^Microbiology and Immunology Department, Faculty of Pharmacy, Heliopolis University, Cairo, Egypt; ^2^Biomedical Research Lab, Research and Development Department, Faculty of Pharmacy, Heliopolis University, Cairo, Egypt; ^3^Pharmaceutical Division, Ministry of Health and Population, Faiyum, Egypt; ^4^Microbiology and Immunology Department, Faculty of Pharmacy, Al-Azhar University, Cairo, Egypt

## Abstract

The emergence of *AmpC* (*pAmpC*) *β*-lactamases conferring resistance to the third-generation cephalosporins has become a major clinical concern worldwide. In this study, we aimed to evaluate the expression of *AmpC β*-lactamase encoding gene among the pathogenic Gram-positive and Gram-negative resistant bacteria screened from clinical samples of Egyptian patients enrolled into El-Qasr El-Ainy Tertiary Hospital in Cairo, Egypt. A total of 153 bacterial isolates of the species *Pseudomonas aeruginosa*, *Klebsiella pneumoniae,* and *Enterococcus faecium* were isolated from patients diagnosed with urinary tract infection (UTI), respiratory tract infection (RTI), and wound infections. The total number of *E. faecium* isolates was 53, comprising 29 urine isolates, 5 sputum isolates, and 19 wound swab isolates, whereas the total number of *P. aeruginosa* isolates was 49, comprising 27 urine isolates, 7 sputum isolates, and 15 wound swab isolates, and that of the *K. pneumoniae* isolates was 51, comprising 20 urine isolates, 25 sputum isolates, and 6 wound swab isolates. Our results indicated that there is no significant difference in the expression of *AmpC β*-lactamase gene among the tested bacterial species with respect to the type of infection and/or clinical specimen. However, the expression patterns of *AmpC β*-lactamase gene markedly differed according to the antibacterial resistance characteristics of the tested isolates.

## 1. Introduction

The introduction of antibiotics to the clinical practice in the twentieth century to combat infectious diseases and microbes was indisputably a blessing to human civilization that has saved billions of people. However, antimicrobial resistance has steadily been increasing during the last decades and has become a global causative threat to human morbidity and mortality [1]. In the USA, more than 63,000 patients die every year because of hospital-acquired bacterial infections. Similarly, an estimation of 25,000 patients die yearly in Europe, due to infections with multidrug-resistant (MDR) bacteria. The widespread and incautious use of antibiotics has significantly contributed to the advent of resistant microbial strains [2]. Resistance to antimicrobials such as the third-generation cephalosporins, quinolones, and carbapenems continues, particularly, to increase sharply among Gram-negative bacterial pathogens [3].

According to the World Health Organization (WHO) global priority list of antimicrobial-resistant bacteria published in 2017, *Pseudomonas aeruginosa*, *Klebsiella pneumoniae,* and *Enterococcus faecium* are among the common healthcare-associated pathogens of critical or high priority [4]. *Pseudomonas aeruginosa* is an aerobic Gram-negative bacterium that can cause infections in hospitalized patients, as well as in immunocompromised hosts and patients with cystic fibrosis. Therefore, it is one of the worldwide leading nosocomial pathogens that can cause serious nosocomial infections such as pneumonia, urinary tract infections (UTI), bloodstream infections (BSI), surgical-site infections. and skin infections [5]. It can also cause community-acquired infections including ulcerative keratitis, otitis externa, and skin and soft-tissue infections [6]. It is among the increasingly reported and commonly identified multidrug-resistant or even pan-drug-resistant bacteria [5]. *Klebsiella pneumoniae* is a type of Gram-negative bacteria associated with a wide spectrum of infections, such as UTI, pneumonia, intra-abdominal infections, BSI, meningitis, and pyogenic liver abscesses (PLA) [7], whereas *Enterococcus faecium* is a Gram-positive bacterium that began to emerge as a leading cause of multidrug-resistant hospital-acquired infections in the 1970s and 1980s, and it currently ranks among the leading causes of hospital-acquired infections of the bloodstream, urinary tract, surgical wounds, and other sites. It is also found to accompany the obligate anaerobes in mixed infections which results in intra-abdominal abscesses [8].

*AmpC*-type enzymes are *β*-lactamases that mediate resistance to cephalothin, cefazolin, cefoxitin, most penicillins, and *β*-lactamase inhibitor-*β*-lactam combinations [9]. These enzymes may be either encoded on the chromosomes of different microorganisms including many members of the Enterobacteriaceae family or on transmissible plasmids that harbor genes encoding *AmpC* enzymes in bacteria lacking or poorly expressing the chromosomal ones [10]. Carbapenems can usually be used to treat infections caused by *AmpC*-producing bacteria. However, carbapenem resistance may arise in some microorganisms by mutations that reduce influx (outer membrane porin loss) or enhance efflux (efflux pump activation) [10]. *AmpC β*-lactamases can barely degrade carbapenems, but they can covalently bind them to the periplasm and prevent them from accessing their targets [11]. In Egypt, antimicrobial resistance to third-generation cephalosporins is on the rise, causing nosocomial outbreaks. However, a few local studies analyzed the underlying genes behind these increasing resistances. Hence, in our study, we aimed to solve the dilemma of antibiotic resistance through evaluating the role of the expression of *AmpC β*-lactamase encoding gene among selected Gram-positive and Gram-negative MDR-resistant bacteria that commonly cause infections to patients enrolled into El-Qasr El-Ainy Tertiary Hospital in Cairo, Egypt. The MDR bacterial pathogens we selected were *P. aeruginosa*, *K. pneumoniae,* and *E. faecium*. They are categorized in the WHO global priority list of antimicrobial-resistant bacteria as pathogens with critical priority (*P. aeruginosa* and *K. pneumoniae*) or with high priority (*E. faecium*) [4]. In addition, the correlation between the *AmpC β*-lactamase expression and the type of different bacterial strains (Gram positive or Gram negative) and their isolation sources, as well as the correlation between the *AmpC β*-lactamase expression genotype and *AmpC β*-lactamase enzyme phenotype, was investigated.

## 2. Materials and Methods

**Figure 1:**
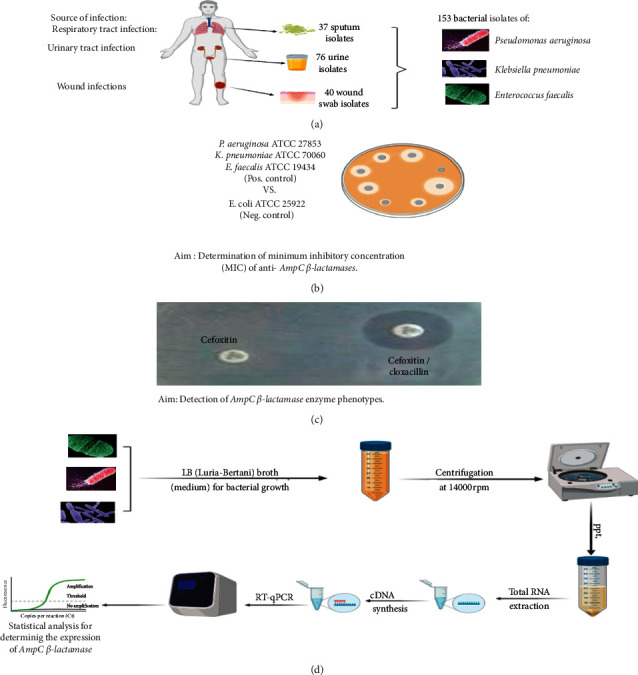
Flowchart of the followed methodology in this study, drawn by BioRender (https://biorender.com/). (a)Bacterial isolates and clinical samples. (b)Antimicrobial susceptibility testing. (c)Cefoxitin-cloxacillin double-disc synergy test (CC-DDS). (d)Quantification of *AmpC β*-lactamase encoding gene expression.

The experimental design of the current study is summarized in Figure 1.

### 2.1. Bacterial Isolates and Clinical Samples

This study was conducted following the Declaration of Helsinki and approved by the Ethics Committee of Heliopolis University and El-Qasr El-Ainy Hospital. Written informed consent was obtained from all participants. The samples were collected from December 2016 to January 2018 from hospitalized patients in El-Qasr El-Ainy Hospital. A total of 153 bacterial isolates obtained from the clinical samples were identified to be of the species *P. aeruginosa*, *K. pneumoniae,* and *E. faecium*. Bacterial isolates were identified to the species level using the conventional microbiological methods (cultural characteristics), and identification was confirmed using the Vitek 2 system (bioMérieux; Marcy l'Etoile, France).

These isolates were recovered from diverse clinical samples including urine, sputum, and wound swabs collected from patients suffering from urinary tract infections (UTIs), respiratory tract infections (RTIs), and wound infections, respectively. The total number of *E. faecium* isolates was 53, comprising 29 urine isolates, 5 sputum isolates, and 19 wound swab isolates, whereas the total number of *P. aeruginosa* isolates was 49, comprising 27 urine isolates, 7 sputum isolates, and 15 wound swab isolates. The total number of *K. pneumoniae* isolates was 51, comprising 20 urine isolates, 25 sputum isolates, and 6 wound swab isolates.

### 2.2. Antimicrobial Susceptibility Testing

Antimicrobial susceptibility testing and minimum inhibitory concentration (MIC) determination using diverse antimicrobial classes were performed according to standard protocols, guidelines, and interpretive criteria described by the Clinical and Laboratory Standards Institute [12]. The standard strains *P. aeruginosa* ATCC 27853, *K. pneumoniae* ATCC 70060, and *Enterococcus faecium* ATCC 19434 (American Type Culture Collection, Manassas, VA, USA) were used as positive controls for the production of *AmpC β*-lactamase. *E. coli* ATCC 25922 was used as a negative control for the production of *AmpC β*-lactamase.

### 2.3. Detection of *AmpC β*-Lactamase Enzyme Phenotypes

#### 2.3.1. Cefoxitin-Cloxacillin Double-Disc Synergy Test (CC-DDS)

This test was used to determine the production of *AmpC β*-lactamase enzyme in 111 isolates. Briefly, 0.5-McFarland suspensions of the test isolate were prepared from overnight cultures. Then, the adjusted suspensions were used to inoculate Muller-Hinton agar plates using sterile swabs. Discs containing 30 *µ*g of cefoxitin plus 200 *µ*g of cloxacillin (inhibitors of the *AmpC* enzyme) were added on the surface of the Muller-Hinton agar. Plates were finally incubated at 35°C for 16–18 hours. A difference in the cefoxitin-cloxacillin inhibition zones minus the cefoxitin alone zones of ≥4 mm was considered indicative for *AmpC* production [13–15].

### 2.4. Quantification of *AmpC β*-Lactamase Encoding Gene Expression

#### 2.4.1. Total RNA Extraction

Bacterial isolates were inoculated into 5 mL aliquots of the LB (Luria-Bertani) broth medium and incubated overnight with shaking (200 rpm) at 37°C. Next, isolates were subcultured in 50 mL aliquots of the LB broth medium and allowed to grow until they reached an optical density (OD600 nm) of 0.6—0.8; then, the cell pellets were harvested by centrifugation at 14000 rpm. The total RNA was extracted from each isolate using the GeneElute bacterial total RNA purification kit (Sigma Aldrich, USA) and eluted in RNase-free water following the manufacture's protocol. The DNA contaminants were eliminated by using 2 U of DNase I, RNase-free enzyme (Puregene, India). RNA concentration and purity were determined using the NanoDrop instrument (Nano-100, UK). The final RNA concentrations extracted from *P. aeruginosa*, *K. pneumoniae,* and *E. faecium* were 10 *µ*g, 15 *µ*g, and 12 *µ*g, respectively, and the purity measured by OD 260/280 ratios was between 1.7 and 1.9 [16].

#### 2.4.2. cDNA Synthesis and Quantitative Real-Time-PCR (RT-qPCR) Experiments

Complementary DNA (cDNA) was synthesized using the bacterial isolates extracted RNA template as follows. Firstly, 30 ng of the purified RNA was subjected to reverse transcription into cDNA using the HisenscriptTmRH [−] cDNA Synthesis Kit (iNtRON Biotechnology, Korea) using a PCR thermocycler (A & E Lab, UK). Then, 5 *µ*l of the cDNA was used for real-time PCR amplification using the Maxima SYBR Green/ROX qPCR Master Mix (2X) (ThermoFisher Scientific, USA) with the StepOne Plus real-time PCR system (Applied biosystem, USA) [17]. All PCRs were performed in duplicate. The specific primers of genes were designed by the primer designing tool at the NCBI (https://www.ncbi.nlm.nih.gov/tools/primer-blast/) and synthesized by HVD life sciences, Cairo, Egypt. The genomic sequences of *K. pneumoniae* (GenBank accession no. EF125013.1) and *E. faecium* (GenBank accession no. NC_017960.1) were used as templates in designing the PCR primers. The oligonucleotide primers used in PCR amplifications are listed in Table 1. Bacterial isolates were considered as positive *AmpC*-producing bacteria if the mRNA levels of *P. aeruginosa*, *K. pneumoniae,* and *E. faecium* were at least 15-fold, 12-fold, and 10-fold overexpressed compared to the control strains.

### 2.5. Statistical Analysis

GraphPad Prism software (version 6.01) was used to perform statistical analysis. The quantitative statistical analyses were performed using one-way ANOVA to determine if there was a significant difference in the *AmpC* expression levels among the three resistant bacterial strains or the sample sources (urine, sputum, and wound). The Spearman correlation test, one tail at *p* < 0.05, was performed to test for correlation between the *AmpC* expression level and antimicrobial resistance. A *p* value of <0.05 was considered statistically significant.

## 3. Results

### 3.1. Antimicrobial Susceptibility Profiles

**Figure 2:**
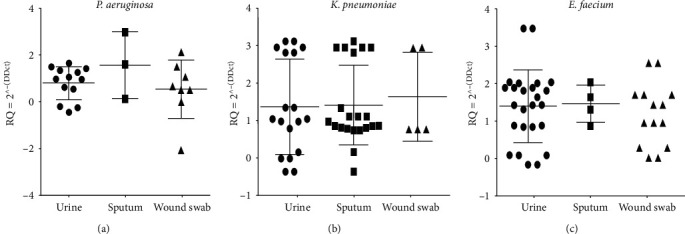
*AmpC β*-lactamase expression pattern, evaluated by RT-qPCR, among the isolates of bacterial species included in this study recovered from urine, sputum, and wound swab.

The antimicrobial susceptibility study showed that there is an overall high resistance of the bacterial isolates included in this study to *β*-lactam antibiotics (including ampicillin, amoxicillin, and piperacillin whether alone or in combination with *β*-lactamase inhibitors), as well as to cephalosporins. The antimicrobial resistance profiles of isolates are listed in Table 2. The recovered bacterial isolates showed high levels of multidrug resistance to the tested antimicrobial agents, as 93 out of the 153 isolates (60.8%) were resistant to three or more classes of antimicrobial agents and described as MDR. Moreover, 23 out of the 153 isolates (15%) were found to be susceptible to only one or two categories of antimicrobials and classified as extensively drug-resistant (XDR) bacterial isolates. For the detection of *AmpC* enzyme production, a CC-DDS phenotypic test revealed that 81% (43/53) of *E. faecium* isolates, 86% (44/51) of *K. pneumoniae* isolates, and 48.9% (24/49) of *P. aeruginosa* isolates were *AmpC*-producing phenotypes. The evaluation of *AmpC β*-lactamase encoding gene expression, by real-time PCR detection of *AmpC* transcript, showed that the gene is expressed in 81% (43/53) of *E. faecium* isolates and 86% (44/51) and 48.9% (24/49) of *P. aeruginosa* isolates (Figure 2 and Table 3).

### 3.2. Quantification of *AmpC β*-Lactamase Encoding Gene Expression

Based on the expression fold change, *AmpC β*-lactamase gene was expressed in a total of 54 (48.65%) isolates comprising 13 (54.17%) *P. aeruginosa* isolates, 19 (43.18%) *K. pneumoniae* isolates, and 22 (51.16%) *E. faecium* isolates (Tables 2 and 4).

**Figure 3:**
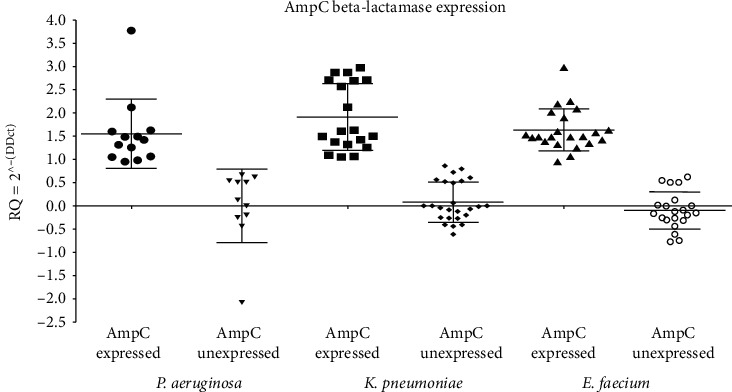
*AmpC β*-lactamase expression evaluated by RT-qPCR among bacterial species included in this study.

The fold change of the expression of the *AmpC β*-lactamase gene among the isolates of *P. aeruginosa*, *K. pneumoniae,* or *E. faecium* did not show any significant difference in their expression levels regardless of being from the urine, sputum, or wound swab origin, *p* > 0.05, as shown in Figure 2 and Table 3. The difference in the fold change of the expression of the *AmpC β*-lactamase encoding gene in the isolates of each bacterial species with *AmpC* expressed and those with *AmpC* unexpressed was revealed to be statistically significant indicated by three asterisks at the *p* value 0.0001 (Figure 3 and Table 3). However, it was found that the level of expressed *AmpC β*-lactamase gene within the three different species showed a nonsignificant difference of *p* > 0.05.

## 4. Discussion

Antibacterial resistance (ABR) is the insensitivity of bacteria to antibacterial drugs, thus causing difficulty in the treatment of infectious diseases and, consequently, high mortality. *AmpC β*-lactamase, produced by the *AmpC* gene, is the first determined destroyer of the *β*-lactam ring of *β*-lactam antibiotics, resulting in the phenomenal challenge of ABR. Thus, the phenotypic and genotypic aspects of the *AmpC* gene concerning ABR are interesting to study [19, 20]. It has been shown that *AmpC β*-lactamase has potential repressing activities on a wide range of antibacterial agents. Indeed, it was reported that the resistance to drugs such as cephalothin, cefazolin, cefoxitin, and most penicillins was epidemiologically and genetically related to the expression of the *AmpC* gene in the bacterial species *P. aeruginosa*, *K. pneumoniae,* and *E. faecium* [21–26]. Growing evidence shows that antimicrobial resistance towards third-generation cephalosporins is increasingly outbreaking in community hospitals that, in some cases, especially with Enterobacteriace infections, can cause mortality [27]. However, limited research investigated the causes. Therefore, in the current study, we aimed to evaluate the impact of *AmpC* expression on the resistance to different antibacterial agents among clinical isolates of three healthcare-associated pathogens collected from a tertiary hospital in Cairo, Egypt, namely, two Gram-negative (*P. aeruginosa* and *K. pneumoniae*) and one Gram-positive (*E. faecium*) bacteria with critical or high-priority classification, respectively, in the WHO global priority list of antimicrobial-resistant bacteria [4], and based on the bacterial strain and source of infection; three different sources of infection were used to recover the bacterial isolates. These sources were bacterial UTIs, RTIs, and wound infections. Contextually, the worldwide prevalence of bacterial UTIs is 11% [28] and that of RTIs is 5–15%, secondary to influenza virus infections [29].

As for the worldwide prevalence of the underinvestigated bacterial strains related to the source of infection, a study covering 3193 patients in 54 countries with confirmed diagnosis of community-acquired pneumonia (CAP) reported that *P. aeruginosa* was responsible for 4.2% and 2.0%, respectively, of the *P. aeruginosa*- and antibiotic-resistant *P. aeruginosa*-CAP infections. The critical rate of *P. aeruginosa* causing RTIs reached 67% of patients suffering from tracheostomy, bronchiectasis, and/or very severe chronic obstructive pulmonary disease [30]. In the UTIs/wound infections, *P. aeruginosa* was presented as the third causative factor in UTIs by 12% and wound infections by 29.6% [31]. For *K. pneumoniae*, it is prevalently responsible for 15–40%/32.6% of pneumonia/UTIs cases, respectively [30]. It is reported that *E. faecium* is prevalently responsible for 15% of infections commonly caused by enterococci such as UTIs, RTIs, and wound infections [32–34].

In the current study, the expression of the *AmpC β*-lactamase gene investigated by the expression patterns of the *AmpC β*-lactamase gene, in resistant and nonresistant isolates, was analyzed using RT-qPCR experiments in 153 clinical isolates of *P. aeruginosa*, *K. pneumoniae,* and *E. faecium* (Table 4). Results revealed that there was no significant difference in *AmpC* overexpression among the tested bacterial species, regardless of their Gram ± phenotype, type of infection, or clinical specimen.

This is a confirmation of what was previously reported in [11] that in GenBank, *AmpC* genes are included in COG 1680, where COG stands for a cluster of orthologous groups. COG 1680 comprises other penicillin-binding proteins as well as class C *β*-lactamases and includes proteins from archaea as well as bacteria, Gram-positive as well as Gram-negative organisms.

Our study demonstrated that the expression of the *AmpC* gene was detected in a total of 54 (48.65%) isolates of *P. aeruginosa*, *K. pneumoniae,* and *E. faecium* with no significant difference in the *AmpC* expression level concerning the type of infection or the source of specimen, urine (*AmpC* expression of 50.45% where *n* = 56), sputum (*AmpC* expression of 25.23% where *n* = 28), or wound swab (*AmpC* expression of 24.32% where *n* = 27). These findings are consistent with the results of another study from Egypt regarding the expression of *AmpC β*-lactamase in microorganisms obtained from Ain Shams University Hospital of Egypt, indicating that 34.8% (22/46) isolates of *K. pneumoniae* showed overexpression of the *AmpC* gene [35, 36]. We found that 60% of isolates tested in this study were MDR, which is along the same line of another study conducted in Egypt, and found that 87.5% of the clinical isolates were MDR [37]. Indeed, isolates recorded as *AmpC* positive using the cefoxitin disc test showed resistance to diverse antimicrobial classes including cephalosporins, ciprofloxacin, amikacin, levofloxacin, cefoperazone-sulbactam, piperacillin/tazobactam, meropenem, and imipenem [38]. Moreover, there was no significant difference in the *AmpC* expression level in comparison to antibacterial resistance to the abovementioned antibiotics using the Spearman correlation, *p* > 0.05. That means that there may be other genes which can play an important role together with the *AmpC* gene in the antibiotic resistance.

For ABR by *K. pneumoniae,* efforts were put to find the temporal seasonal correlation between its ABR and misuse of *β*-lactam/*β*-lactamase inhibitors (BLBLIs), so statistical analysis has been performed by another group to correlate the annually prescribed antibiotics at a tertiary-care hospital of Korean Health Insurance to the detected ABR cases. They found that replacing carbapenems with BLBLIs is not the ideal solution, as ABR is likely to spread rapidly against the piperacillin/tazobactam combination [39]. Also, it was indicated that *β*-arrestin recruiting gene is a crucial inducer of the *β*-lactamase signaling pathway, increasing the ABR of *K. pneumoniae* against clindamycin, erythromycin, linezolid, and penicillin [40].

For *P. aeruginosa*, our findings are consistent with those of another study which revealed that *AmpC*-positive *P. aeruginosa* isolates obtained from burn patients exhibited resistance to cephalosporins and carbapenems [41]. The underlying molecular factors of *AmpC* positivity in *P. aeruginosa* were illustrated as the occurrence of genetic mutations in the gene bla*AmpC* which encodes a wide-spectrum class C *β*-lactamase and AmpR-activating mutation (G154R), causing an extended-spectrum *AmpC*s resistance to ticarcillin and piperacillin, monobactams (aztreonam), and third-generation (ceftazidime) and fourth-generation (cefepime) cephalosporins [42, 43]. An extended screening study in northeast China reported that the high prevalence of MDR *P. aeruginosa* strains exhibiting resistance to ticarcillin/clavulanic acid, piperacillin/tazobactam, cefoperazone/sulbactam, piperacillin, imipenem, meropenem, ceftazidime, cefepime, ciprofloxacin, and levofloxacin is significantly correlated to infections occurring after hospital admission [44]. Moreover, a retrospective study that investigated the development of resistance to antimicrobials in *P. aeruginosa* showed that imipenem or ciprofloxacin therapy is a key factor of producing resistant strains of *P. aeruginosa* [45]. Similarly, it was indicated that the ABR of *E. faecium* to *β*-lactams is correlated with possessing the blaZ-blaI-blaR1 operon [46].

Many genes are speculated to be involved in the complicated antibiotic resistance characteristics of microorganisms, but the data available are limited. The genetic clarification that the hyperproduction of *AmpC* increases the resistance to *β*-lactams is corroborated by the diarrheagenic *E. coli* (DEC) studies. Based on the transcriptome analysis of *AmpC β*-lactamases isolated from DEC, it was reported that mutational changes occurred within the *AmpC β*-lactamase genes resulting in its increased transcription [47]. Moreover, it was indicated that the mutations of *β*-N-acetylglucosaminidase and PG recycling enzyme play a crucial role in enhancing the *AmpC* expression via interaction with ampR, the regulatory gene of *AmpC*, additionally to the plasmid-carrying *AmpC* gene as a source of *AmpC* overexpression [43, 48]. It was reported, in the case of drug-sensitive isolates, that the mRNA expression levels were mostly found downregulated, except for few isolates where they were comparable to the control strains, suggesting their nonpathogenic nature. However, the downregulation in sensitive strains could be a result of the process of genetic gain or development. Constitutive expression of *AmpC* occurs at a lower level in *E. coli* [49, 50]; however, various mutations may result in constitutive overexpression of the *β*-lactamase-producing genes [51–53].

To uncover the extent of the remarkable crisis of *AmpC* impact on ABR, we investigated the antimicrobial resistance patterns of the screened isolates and correlated them with the phenotypic overexpression of *AmpC*. Our results showed that the isolates of the diverse bacterial species included in the study, regardless of their source of infection, were 100% resistant to ampicillin, amoxicillin, amoxicillin-clavulanate, ampicillin-sulbactam, piperacillin, piperacillin-tazobactam, and cefaclor. Overall, the bacterial isolates showed a multidrug-resistance rate of 60.8% and an extended drug resistance of 15%. Consistently, these findings owe positively phenotypic correlations to the CC-DDS test whose results for *AmpC* are 81% (43/53) for *E. faecium* isolates, 86% (44/51) for *K. pneumoniae* isolates, and 48.9% (24/ 49) for *P. aeruginosa* isolates. From these findings, it is evident that 100% of the tested isolates show ABR to a broad number of antimicrobials. Moreover, we observed that only 48.65% of the isolates showed an expressed *AmpC* gene, suggesting that *AmpC* is not the only factor responsible for ABR and that the phenomenal ABR is a complex process involving multigenetic factors.

To tailor an effective solution for the dilemma of ABR, it is recommended to possess a comprehensive understanding of the biological causes of ABR by *β*-lactams, design novel therapies for microorganisms exhibiting ABR based on our understanding of its underlying factors, and shed light on the widespread antibiotic misuse. The known causes of ABR may be summarized as follows: (i) showing resistance to the bacterial cell wall active agents such as *β*-lactams (e.g., ampicillin and penicillin) which exert an inhibiting activity on the peptidoglycan synthesis of the cell wall, as in *E. faecium*. This resistance property may be rendered by mutated overexpressed pbp5 (Met485 ⟶ Ala) and LDTfm, resulting in lowered binding affinity of the *β*-lactams to the bacterial cell wall, thus allowing the peptidoglycan synthesis [54, 55]. (ii) Drug inactivation: the *β*-lactam ring is directly susceptible to cleavage by *β*-lactamase enzymes encoded by the gene blaZ which inactivates antibiotics [56]. (iii) Cell signaling/resistance by mutation: acquired mutations of the intrinsic genes in *E. faecium* are crucial factors causing ABR. Mutated CroRS [57, 58], IreP, and IreK cause resistance to cephalosporins [39]. In this respect, it was revealed that *β*-arrestin is a master player for *K. pneumoniae* to generate resistance to *β*-lactams [59]. For *P. aeruginosa*, deficiency in oprD porin results in developing resistance to carbapenems. Additionally, resistance of *P. aeruginosa* to *β*-lactams is due to the intrinsic mutated genes (mexR, nalB, nalC, or nalD)/nfxB as key factors for overexpression of MexAB-OprM/MexCD–OprJ, respectively [39, 53, 60, 61]. Based on the antimicrobial susceptibility profiles in the current study, it is strongly hypothesized that *K. pneumoniae*, *P. aeruginosa,* and *E. faecium* isolates have multicausative factors for ABR. Hence, these isolates showed similar resistance patterns to the tested antimicrobials.

It is worth mentioning that the critical question of “how these abovementioned resistance-related genes, either in sequence or in expression, result in ABR?” is not fully answered, and the underlying mechanisms are not clearly understood. The proposed answer is based on knockdown assays to uncover the phenotype of genetic functions of the bacterial susceptibility to antibiotics [62–64]. Moreover, the global health issue of antibiotic misuse is an important driving reason to ABR, as antibiotic misuse evolves hypermutable bacteria through the natural selection empowerment against antibiotics, mediated by the transfer of transmissible resistance (R) plasmids [65] through bacterial cell walls of different microorganisms as carriers of genes encoding *β*-lactamase enzymes [63].

Besides, it is worth stating that antibacterial vaccines, based on bacterial pathogenesis and antiviral vaccines, for the viruses causing a secondary bacterial infection are a blessing that results in the reduction of prescribed antibiotics [66, 67]. As potentially solving approaches for ABR, (i) it is by replacing antibiotics with phage therapy [68], (ii) phage therapy owes significant specificity to bacteria than antibiotics and controllable to be biologically designed against wide bacterial spectrum, (iii) CRISPR-Cas9 is a promising biotechnological tool to abolish pathogenically bacterial genes causing their ABR, especially for children and elders [69, 70], and (iv) further studies for comprehensive understanding of the molecular biology behind *AmpC* overexpression are required to develop new drugs for resistant infections.

Our work is an addition to the field of bacterial infection therapeutics, aiming to oppose the common habit of antibiotic misuse [65] by healthy individuals, to avoid the emergence of resistant microorganisms. We recommend the performance of further studies to discover new therapeutic alternatives other than the antibiotic-monotherapy for different bacterial infections.

## Figures and Tables

**Table 1:** PCR oligonucleotide primers used for the RT‐qPCR amplification of the *AmpC* gene extracted from the bacterial species of our study.

Bacterial species	Primer name	Sequence (5′—3′)	PCR product	Reference
*P. aeruginosa*	PA. *AmpC*-F	CGCCGTACAACCGGTGAT	113 bp	[18]
	PA. *AmpC*-R	GAAGTAATGCGGTTCTCCTTTCA		
*K. pneumoniae*	KP. *AmpC*-F	ATCTGGCAACCTATACCGCA	227 bp	This study
	KP. *AmpC*-R	CTTGAGCGGCTTAAAGACCC		
*E. faecium*	EF. *AmpC*-F	GATCGACAGGATGTACGCGA	300 bp	This study
	EF. *AmpC*-R	CAGGTAACGCGGGTCTCTTT		

**Table 2:** Antimicrobial resistance patterns of isolates included in this study.

Antimicrobial agent^*∗*^	*E. faecium* (53 isolates) *N* (%)^*∗∗*^	*K. pneumoniae* (51 isolates) *N* (%)^*∗∗*^	*P. aeruginosa* (49 isolates) *N* (%)^*∗∗*^
AMP	53 (100)	51 (100)	49 (100)
AMX	53 (100)	51 (100)	49 (100)
AMC	53 (100)	51 (100)	49 (100)
SAM	53 (100)	51 (100)	49 (100)
PIP	53 (100)	51 (100)	49 (100)
TZP	53 (100)	51 (100)	38 (77.6)
CEC	53 (100)	51 (100)	49 (100)
CFR	53 (100)	51 (100)	49 (100)
FEP	26 (49)	39 (78)	27 (55)
CFP	38 (72)	44 (86)	30 (61)
CTX	10 (19)	45 (88)	27 (55)
CAZ	53 (100)	45 (88)	40 (82)
CRO	11 (21)	45 (88)	41 (84)
CXM	11 (21)	45 (88)	39 (80)
MEM	26 (49)	36 (71)	18 (37)

^*∗*^AMP, ampicillin; AMX, amoxicillin; AMC, amoxicillin-clavulanate; SAM, ampicillin-sulbactam, TZP, PIP, piperacillin; piperacillin-tazobactam; CEC, cefaclor (30 *μ*g); CFR, cefadroxil; FEP, cefepime; CFP, cefoperazone; CTX, cefotaxime; CAZ, ceftazidime; CRO, ceftriaxone; CXM, cefuroxime (axetil or sodium); MEM, meropenem. ^*∗∗*^Percentage of resistant isolates correlated to the total number of isolates within each bacterial species (*N*).

**Table 3:** Mean fold change of *AmpC* expression among the isolates of bacterial species isolated from urine, sputum, or wound swab samples and included in this study.

Bacterial species and mean fold of *AmpC* expression		Clinical specimen			*p* value
		Urine	Sputum	Wound swab	
*P. aeruginosa*	Mean fold change of *AmpC* expression ± SE	0.799 ± 0.194	1.57 ± 0.821	0.538 ± 0.44	0.3278
	Number (%)	13 (54.17%)	3 (12.50%)	8 (33.33%)	

*K. pneumoniae*	Mean fold change of *AmpC* expression ± SE	1.12 ± 0.3	1.17 ± 0.232	1.39 ± 0.531	0.9004
	Number (%)	18 (40.91%)	21(47.73%)	5 (11.36%)	

*E. faecium*	Mean fold change of *AmpC* expression ± SE	1.03 ± 0.908	1.09 ± 0.461	0.835 ± 0.787	0.9869
	Number (%)	25 (58.14%)	4 (9.30%)	14 (32.56%)	

Total		56 (50.45%)	28 (25.23%)	27 (24.32%)	

**Table 4:** Mean fold change in differential expression of *AmpC* (*AmpC* expressed vs. *AmpC* unexpressed) among the bacterial species included in this study.

Bacterial species	Number (%)		Mean fold change of *AmpC* expression ± SE		*p* value
	*AmpC* expressed	*AmpC* unexpressed	*AmpC* expressed1	*AmpC* unexpressed	
*P. aeruginosa*	13 (54.17%)	11 (45.83%)	1.552 ± 0.206	0.002 ± 0.239	^*∗∗∗*^0.0001
*K. pneumoniae*	19 (43.18%)	25 (56.82%)	1.913 ± 0.165	0.081 ± 0.086	^*∗∗∗*^0.0001
*E. faecium*	22 (51.16%)	21 (48.84%)	1.633 ± 0.096	−0.100 ± 0.087	^*∗∗∗*^0.0001
Total	54 (48.65%)	57 (51.35%)			

## Data Availability

Data used to support this study can be obtained upon request.
